# Adjunctive Immunotherapy With Polyclonal Ig-M Enriched Immunoglobulins for Septic Shock: From Bench to Bedside. The Rationale for a Personalized Treatment Protocol

**DOI:** 10.3389/fmed.2021.616511

**Published:** 2021-02-18

**Authors:** Stefano Busani, Erika Roat, Martina Tosi, Emanuela Biagioni, Irene Coloretti, Marianna Meschiari, Roberta Gelmini, Lucio Brugioni, Sara De Biasi, Massimo Girardis

**Affiliations:** ^1^Intensive Care Unit, University Hospital Policlinico di Modena, University of Modena and Reggio Emilia, Modena, Italy; ^2^Infectious Diseases Unit, University Hospital Policlinico di Modena, University of Modena and Reggio Emilia, Modena, Italy; ^3^Department of Medical and Surgical Sciences, University of Modena and Reggio Emilia, Modena, Italy; ^4^Internal Medicine Department, Azienda Ospedaliero-Universitaria Policlinico of Modena, Modena, Italy; ^5^Department of Medical and Surgical Sciences for Children and Adults, University of Modena and Reggio Emilia School of Medicine, Modena, Italy

**Keywords:** immunoglobulins, septic shock, adjunctive treatment, sepsis, protocol

## Abstract

Septic shock still has a high mortality rate which has not hinted at decreasing in recent years. Unfortunately, randomized trials failed mainly because the septic patient was considered as a homogeneous entity. All this creates a sort of therapeutic impotence in everyday clinical practice in treating patients with septic shock. The need to customize therapy on each patient with sepsis has now become an established necessity. In this scenario, adjuvant therapies can help if interpreted as modulators of the immune system. Indeed, the host's immune response differs from patient to patient based on the virulence of the pathogen, comorbidity, infection site, and prolonged hospitalization. In this review, we summarize the rationale for using immunoglobulins as an adjunctive treatment. Furthermore, we would like to suggest a possible protocol to personalize treatment in the different clinical scenarios of the host's response to serious infectious events.

## Septic Shock: A History of Failing Adjuvant Treatments

Septic shock is a complex syndrome occurring when sepsis is associated with circulatory, cellular, and metabolic abnormalities to such an extent that the risk of death is substantially increased compared to sepsis alone. The clinical criteria to define this condition have recently been modified to improve its identification (1).

Despite the progressive comprehension of its pathogenesis, mortality rates are high and did not significantly change in the last 10 years. Septic shock hospital mortality was described as around 40% in a recent meta-analysis analyzing data from 71 studies from Europe and North America (2).

Another worrying aspect of septic shock is randomized clinical trials (RCTs) designed in the last years to test additional therapies that gave discouraging results. Historically therapeutically strategies, some of them appearing promising in preclinical studies, have been developed based on septic shock pathogenesis.

One of the first targets identified and studied was endotoxin present in gram-negative bacteria, which has been blocked through different anti-lipid A antibodies without obtaining benefit in RTCs (3, 4). Similarly, the use of anti-TNF antibodies or anti-IL-1 antibodies was developed with the purpose to limit the innate immune hyperactivation responsible for tissue damage, but larger RTCs results were negative (5, 6). Endothelial dysfunction, frequently found in septic patients, was investigated trying to improve microcirculation and tissue oxygenation, but neither platelet-activating factor antagonist (7) nor activated protein C (8) reduced mortality.

Several different extracorporeal blood purification techniques have been developed in the last decades to remove inflammatory mediators. High volume hemofiltration was unable to reduce mortality in a recent meta-analysis (9), although was considered a safe technique. Hemoperfusion using filters coated with polymyxin B, aimed to remove endotoxins able to trigger the inflammatory response, displayed contrasting results in RCTs (10, 11). Concerning coupled plasma filtration and adsorption (CPFA) interesting results were obtained in the COMPACT 1 randomized study (12) but the COMPACT 2 trial was stopped earlier because of adverse events associated with CPFA. An urgent letter was sent to all CPFA users mentioning that CPFA is no longer indicated for the treatment of septic shock^1^. Among new membranes, Cytosorb a hemoperfusion cartridge able to remove broad-spectrum cytokines failed to find any decrease of IL-6 plasma levels over time (13); while a recent proof of concept pilot study demonstrated a significant effect on norepinephrine requirements (14). The new Oxyris membrane, a heparin-grafted membrane specifically designed for cytokine and endotoxin adsorption, tested on 16 patients seemed to effectively remove endotoxin and TNF-α, IL-6, IL-8, and IFNγ in patients with septic shock-associated acute renal failure (15).

Further, the unsuccessfully tested approach included immunomodulant and antioxidant therapies, aimed to reduce the overwhelming tissue damage caused by the excessive activation of the host's response. The very recent ACTS and ATESS RCTs failed to demonstrate improvement in organ function with a combination of vitamins and corticosteroids (16, 17). The promising results obtained by Meisel et al. about granulocyte-macrophage colony-stimulating factor in sepsis (18), unfortunately, have not yet found confirmation from other studies with adequate power.

As a consequence of this litany of negative studies, over the years the Recommendations provided by the Surviving Sepsis Campaign (SSC) Guidelines (19) for the management of septic shock have been progressively reduced, moreover being in most cases negative (i.e., prescribing not to do something), limiting the therapeutic space of maneuver for clinicians. Thus, although the role of the Guidelines is guided by the “not to harm” principle, stratification into subpopulations may be advisable in a heterogeneous setting.

## Not All Septic Shocks Are the Same. Is It Possible to Tailor Personalized Medicine?

Unfortunately, nowadays it is not easy to answer this question because sepsis is the expression of many different clinical scenarios. Patient characteristics, such as age, comorbidities, genetic factors, immune response, and previous antibiotics exposure are important factors involved in the evolution from sepsis to septic shock; thus, all these variables partly explain the difficulty of defining a unique treatment suitable for all patients.

In light of this observation, some authors developed a clinical staging system called PIRO aimed to characterize septic patients through four components: Predisposition (P), Insult/Infection (I), Response (R), and finally Organ dysfunction (O) evaluating time and number of failing organs. PIRO model can stratify infected patients in 4 stages defined by a progressive increase in mortality trying to reduce the heterogeneity of patients and to help a more tailored therapy (20, 21). Despite this attempt, complexity, and variability have been so far ignored by clinical trials enrolling patients only based on a septic shock diagnosis without considering selecting a specific and more homogeneous subgroup. The lack of patients' selection may in part explain the negative results obtained in all the RCTs so far designed.

The growing comprehension of the essential role played by the immune response in septic shock pathophysiology led to develop and test of some adjunctive treatments based on the use of immunomodulant molecules. To enlighten this fundamental point, it is useful to remember that the immune response during septic shock can be very different among patients. Some patients develop an overwhelming immune response, with a massive production of pro-inflammatory mediators such as cytokines, that are responsible for tissue damage, organ dysfunction, and early death (22). On the other side, some patients develop a condition of immune paralysis, characterized by a reduced ability of the immune system to face pathogens, leading to secondary infections and long-term mortality (22, 23). Generally, mortality in septic shock follows a biphasic curve; only a part of these patients dies during the first 3 days due to an irrepressible immune response, whereas the majority have an unfavorable outcome often a few weeks later, showing a profound impairment of the immune response (24). To confirm this, severe apoptosis-related depletion of cells of both innate and adaptive immune systems along with an increase in regulatory T cells and myeloid-derived suppressor cells, able to inhibit effector immune cell's function, have been documented (23, 25).

Although these scenarios are extreme simplifications of the complex imbalance between activation and suppression of immune response characterizing sepsis and septic shock, it appears clear that agents affecting immune function could be a strategy for implementing sepsis' treatment and improve survival rates.

## The Rationale of Adjunctive Therapy With Polyclonal Intravenous Immunoglobulins

The evidence on Igs, here reported, was found through searches in the Medline (PubMed) and Scopus databases with the January 2000-September 2020 time limits. We narratively reviewed the efficacy and mechanisms of action of Igs, using the keywords “immunoglobulins,” “mechanism of action,” “efficacy” and “sepsis.”

**Figure 1:**
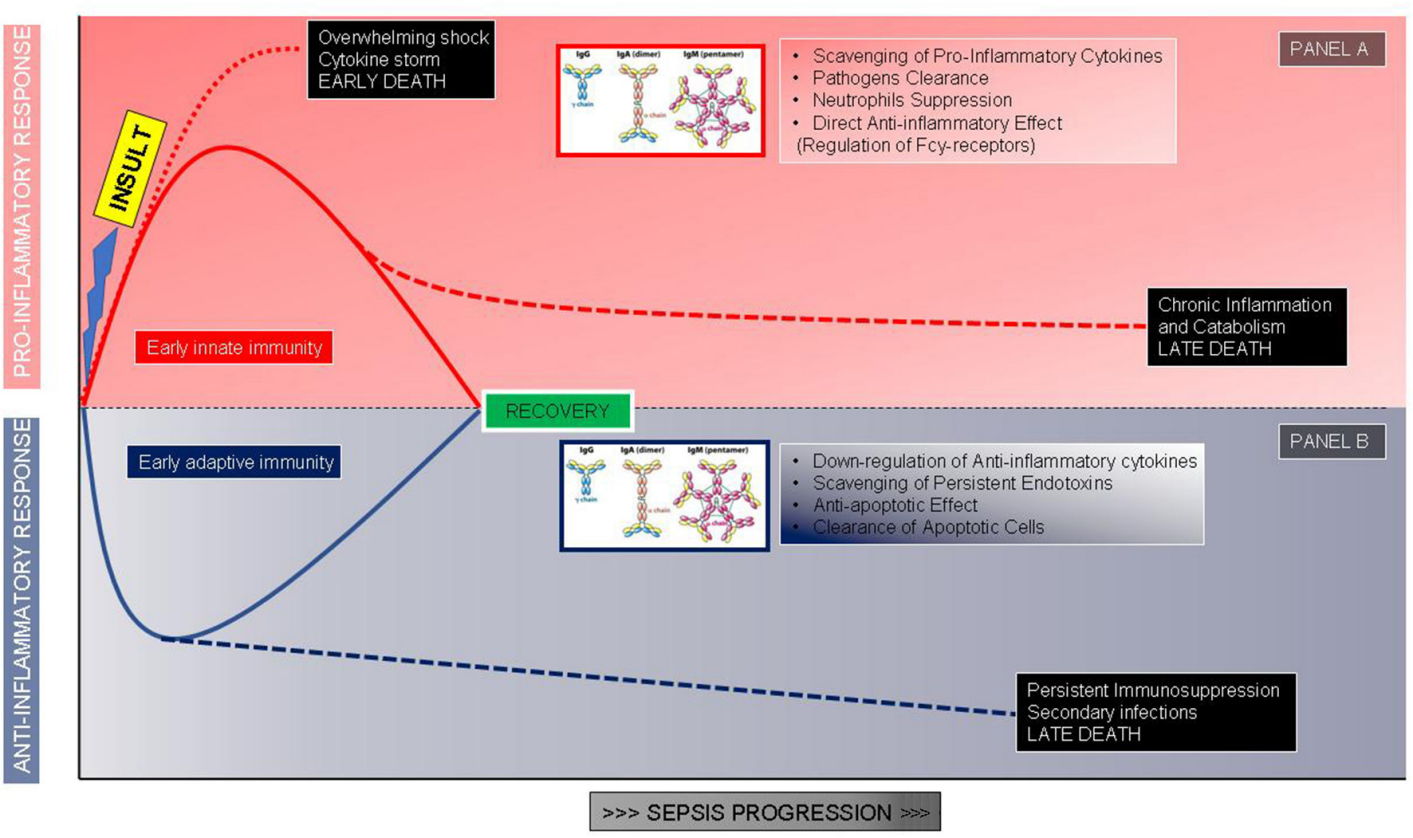
The proposed role of immunoglobulins correlated to the phases of the host response after an infectious insult. **(A)** Hyperinflammatory response and role of immunoglobulins in modulating the proinflammatory storm. **(B)** Immunosuppressive response and role of immunoglobulins as immune system adjuvants.

Among immune-modulatory treatments, intravenous immunoglobulins (Igs) administration may be a promising approach. The rationale for Igs use is both related to their pleiotropic effects on immune response and alterations of Igs serum levels observed in sepsis and septic shock. The potential benefits of Igs administration are related to their immune-modulating functions acting both on pathogens and immune cells: IgG and IgM can scavenge and remove toxins, to bind pathogens promoting phagocytosis, through opsonization and bactericidal activity of CD8+ lymphocytes mediated by antibody-dependent cellular cytotoxicity (26) (Figure 1). These molecules also display direct anti-inflammatory effects mediated by Fc receptor consisting in the modulation of dendritic cell function and reduction in response to INF-γ (27), promote the clearance of apoptotic cells, exert an anti-apoptotic action on immune cells, reduce the activity of the classical complement pathway, due prevalently to IgM, stimulate anti-inflammatory cytokines production along with a reduction of the anti-inflammatory ones favoring a balance between activation and suppression of immune response typically defective during sepsis and septic shock. Igs have also an important synergic activity with antibiotics enhancing their antibacterial efficacy (Figure 1). In addition to the pathophysiological rationale, several studies found a correlation between the reduction of circulating Igs concentrations in septic shock patients and poor outcomes. Venet et al. measured IgG and IgM serum titers in septic shock patients during the first 4 days after diagnosis and found out that Igs deficiency is an important predictive factor for patients' mortality (28). Another study reported an important reduction in IgM levels when patients' conditions deteriorated from severe sepsis to septic shock, underling the importance of Igs kinetic to predict patient's evolution; moreover *in vitro* stimulation with phytohemagglutinin of lymphocytes isolated by septic shock patients displays a reduced ability to produce IgM compared to healthy subjects (29). Despite the relatively high number of studies evaluating this additional therapy (30–36), the scarce number of patients included and the heterogeneity in terms of the type of preparation, dosages, durations, the severity of enrolled subjects, and type of controls impaired the significance of results (Table 1). Nevertheless, two recent meta-analyses revealed a reduction in mortality using polyclonal Igs compared to the control arm and suggested that the highest total dose range is probably more effective (37, 38).

**Table 1:** Description of Igs preparation, dose prescribed, and duration of treatment in different randomized clinical trials in adults evaluating Igs efficacy as adjunctive therapy in sepsis and septic shock.

**Study**	**Type of IG preparation**	**Dose**	**Cumulative dose (g/Kg)**	**Duration (days)**
Toth et al. (30)	IgGAM	5 mL/kg for 3 days	0.75	3
Werdan et al. (31)	IVIG	0.6 g/kg on day 0 and 0.3 g/kg on day 1	0.90	2
Hentrich et al. (32)	IgGAM	1,300 mL for 72 h: 200 mL initially (0.5 mL/min), then 11 infusions of 100 mL every 6 h	0.30	3
Rodríguez et al. (33)	IgGAM	7 ml/Kg daily	1.75	5
Darenberg et al. (34)	IVIG	1 g/kg on day 1 then 0.5 g/kg on day 2 and day 3	2.00	3
Tugrul et al. (35)	IgGAM	5 ml/kg	0.75	3
Karatzas et al. (36)	IgGAM	5 ml/kg	0.75	3

*The evaluated period was January 2000–September 2020. IgGAM, Immunoglobulins G, A, and M; IVIG, polyclonal standard IgG*.

The SSC Guidelines (19) suggested against the use of intravenous Igs in patients with sepsis or septic shock. This conclusion was mainly guided by the results of the only RCT on the use of intravenous Igs judged satisfying in terms of numbers, design, and risk of bias. The study revealed no difference in survival at 28 days, but many limitations reduced its power: long duration from 1991 to 1995, publication only 12 years later, and lack of detail of patients' severity (31). To stress the deficiency of evidence and the lack of agreement among the experts it is important to remind that the last version of the Japanese guidelines for the management of sepsis mentions Igs as a possible therapeutic option (39). At present two different types of preparations obtained from plasma of healthy donors are available: polyclonal standard IgG (IVIG) and IgM-enriched formulation (IgGAM); IVIG contains at least 96% of polyclonal IgG whereas the composition of IgGAM, consisting in IgM 12%, IgA 12%, and IgG 76%, appears to trace more accurately the physiological antibodies' production in course of infection. Both preparations can induce pathogen clearance but the higher killing on gram-negative bacteria is obtained with IgM-enriched Igs (40). The presence of IgM seems to be particularly important because of their specific roles in immune response (29). The characteristics of IgM along with the better results obtained with IgGAM in terms of the odds ratio that is a reduction of mortality's relative risk compared to IVIG in the meta-analysis (41) suggest their preferential use in septic shock. Finally, a recent pilot trial evaluating the use of IgGAM in patients with a diagnosis of sepsis or septic shock observed an improvement in sublingual microcirculation in treated patients (42) suggesting a role in the restoration of endothelial cell function as shown in previous preclinical studies (43). The authors of a recent review evaluating the use of Igs as adjunctive therapies in severe infections in ICU suggest that so far insufficient evidence is available to support their use except for streptococcal toxic shock syndrome. However, they underline the importance to identify the proper Igs type of preparation together with dose, the timing of administration, and patients' characteristics (44). Considering the importance of timing to maximize the effect of immune-modulating therapies, Berlot et al. showed that early administration of IgGAM in septic shock patients was associated with a reduction in the risk of in-ICU mortality (45). A multicenter, double-blind, randomized, controlled trial using hyperimmune Igs to treat patients affected by severe H1N1 infection found that their early use within 5 days of symptom onset was associated with reduced viral load and reduced mortality (46). More recently Yun et al. observed that the administration within 48 h of admission in ICU of intravenous Igs as adjuvant therapy in patients suffering from SARS-CoV-2 pneumonia was associated not only with a reduction in the need for mechanic ventilation but also with a reduction of mortality and in-hospital length of stay (47). Summarizing, we can conclude that there is a pressing need for more precise use of Igs in terms of patients' selection, dosage, and timing rather than excluding their benefit in treating sepsis and septic shock.

## Immunoglobulins' Side Effects

There are no absolute contraindications to the use of Igs. Each product containing Igs is different so a patient with a life-threatening reaction to one product may have no reactions with other preparations. Thus, the contraindications are related to the particular component of the Igs product (48).

Even if in most cases Igs infusions are well-tolerated, several adverse effects have been reported having a wide range of incidence (49); these side effects are more frequently transient but rarely are serious and can lead to long term disability. Two types of risk factors for adverse effects have been identified: one related to Igs preparation and another to the patient's characteristics. Considering Igs formulation a higher concentration of IgA, anti-Rh and anti-RhD increase the prevalence of adverse reactions (50), on the other side some authors reported a greater risk of side effects in patients with primary antibody defects particularly in those with low IgA levels (51), attention should be paid also in patients with a previous history of allergies and thrombotic events. Immediate adverse effects are the majority and consist of flu-like symptoms (which are the most frequent), dermatologic reactions, arrhythmia, hypotension, and transfusion-related acute lung injury (52). On the contrary delayed adverse reactions, although affecting <1% of treated patients, can be severe and in rare cases lethal; these include renal impairment, hematological and neurological disorders, electrolytes alterations, transfusion-related infections, and thrombotic events (52). The incidence of the adverse effects is strictly related to the rate of Igs infusion, therefore during the first administration, it is recommended to start slowly in the first 30 min increasing subsequently the rate (53).

## From Bench to Bedside: A Personalized Protocol for Iggam Adjunctive Treatment

Based on the need to select the correct phenotype for Igs treatment we decided to use “case examples.” The use of these “case examples” could be beneficial to reduce complexity and define some categories of patients uniform in terms of the immune response.

The first “example” is represented by a previously healthy young adult who develops meningococcemia or severe pneumonia sustained by Streptococcus pneumonia or toxic shock syndrome; in this patient, we could expect an overwhelming pro-inflammatory response, not balanced by an adequate anti-inflammatory response, able to eradicate bacteria but leading to tissue damages and multiorgan failure in the early phase of shock (Figure 1A). In this case, we could take advantage of the Igs ability in pathogen clearance, toxin, and mediators scavenging along with the anti-inflammatory effects; the administration should be early and at high dosages to block as soon as possible the hyperactivation of the efficient immune system and limit organ damages (26, 54, 55). Swedish surveillance data on Igs administered in toxic shock syndrome reported a significantly improved survival in treated patients with an odds ratio of 5.6 for Igs use (56). While a very recent prospective multicenter Scandinavian study identified as a risk factor associated with mortality the non-administration of Igs in patients with necrotizing soft-tissue infections (57).

The second “example” is a patient with persisting or breakthrough infection after a first sepsis episode: i.e., candidemia after abdominal surgery or multi-drug resistant (MDR) pathogen infection after a previous infection. The context, in this case, is a reduced pro-inflammatory response combined with a pronounced or sustained anti-inflammatory state with persisting or secondary infections. The patient may manage to limit the pathogen growth, thanks mainly to antibiotic therapy, although a complete clearance of bacteria is not reached, with the persistence of infection leading at a later time to the patient's death (Figure 1B). Another typical example is the appearance of breakthrough infections sustained by opportunistic relatively avirulent pathogens such as Acinetobacter or Stenotrophomonas species. In this condition Igs will be useful thanks to the anti-apoptotic effect on immune cells and the ability to clear apoptotic cells, rather than for pathogen phagocytosis; the time window of administration, in this case, could be broader. Infections caused by opportunistic pathogens, often MDR, are a clear marker of an immune-suppressed status and some evidence suggests the use of IgGAM in this specific condition (26, 55). In our experience on septic shock patients sustained by MDR, preexisting cancer, by itself a condition of immune response impairment, and *Acinetobacter baumanii* infection were independently correlated with an increased risk of 30-days mortality whereas only IgGAM administration appeared to be beneficial (58); similar results were obtained in Greece, a country where the MDR prevalence is extremely high, by Giamarellos-Bourboulis et al. showing, in severe infections by MDR gram-negative bacteria, a protective effect of IgGAM administration on 28-days mortality (59).

**Figure 2:**
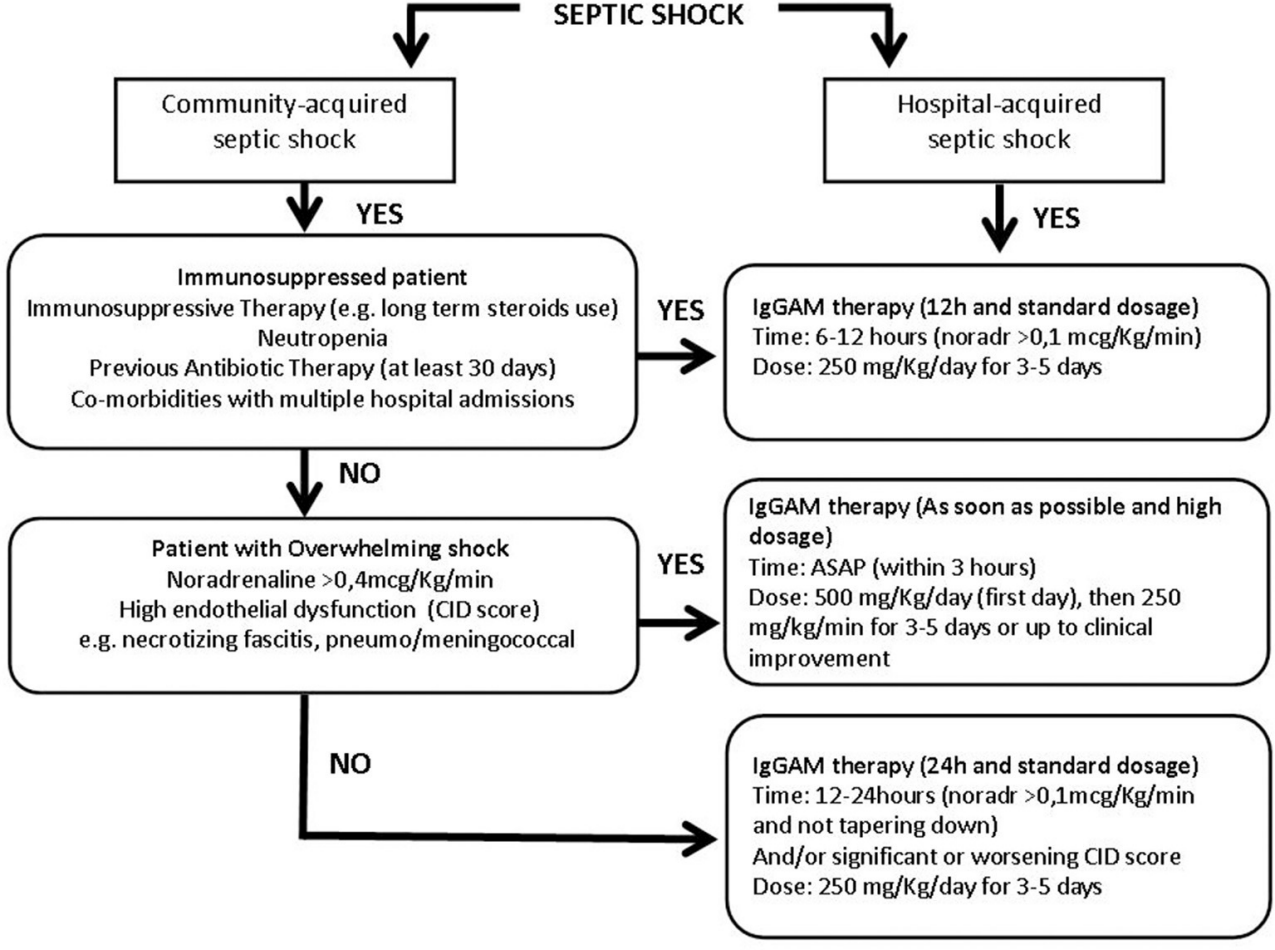
Flow chart of the immunoglobulins' treatment protocol in patients with different septic shock phenotypes. More details in the text. IgGAM, Immunoglobulins G, A, and M; ASAP, as soon as possible; Noradr, Noradrenaline.

To categorize patients and progress toward a more personalized medicine we developed a protocol for IgGAM use in septic shock (Figure 2) aimed at applying this adjunctive therapy to appropriate patients at the right time and dosage.

Community and hospital-acquired septic shocks were first of all distinguished. In the context of the community-acquired infection, the cornerstone is the immediate identification of an overwhelming shock condition, such as necrotizing fasciitis or meningococcemia. Community-acquired septic shocks, developed in non-immunocompromised patients and characterized by the need for high noradrenaline dosages (>0.4 mcg/kg/min) and deep endothelial dysfunction must be promptly identified as overwhelming shock conditions. Endothelial dysfunction may be defined through the CID score, a simple score based on laboratory data: platelet count, fibrinogen, and fibrin markers (i.e., D-dimer or fibrin split products) plasma concentration and prothrombin time, used to identify endothelial alteration and risk of disseminated intravascular coagulation (60). In these conditions, IgGAM therapy should be administered as soon as possible, in any case within 3 h, starting the first day with a higher dose of 500 mg/kg/day (doubling the standard dose) then continuing with 250 mg/kg/day for 3–5 days or up to clinical improvement (Figure 2). The aim is to use IgGAM to scavenge toxins and to improve the clearance of bacteria boosting antibiotic effect and promoting phagocytosis.

If an overwhelming shock is not present, patients coming from the community should be evaluated to identify a possible immune-suppressed status: a previous antibiotic exposure in the last 30 days, neutropenia, defined by a neutrophil count <1,000/mm^3^, or on immunosuppressive therapy, including long term use of corticosteroids are even individually sufficient to define a condition of immune-depression. In this case, Igs administration should be started within 6–12 h from shock occurrence, when noradrenaline dosages are higher than 0.1 mcg/Kg/min. IgGAM in that instance should be used at a standard dose of 250 mg/Kg/day for 3–5 days (Figure 2); we don't need a high dose to control an excessive cytokine release or to reduce the bacterial burden, but we take advantage of the immune support given by the treatment, being usually endogenous Igs reduced. In this case, the goal is to favor the balance between pro and anti-inflammatory response thanks to the Igs' role in modulation of pattern recognition receptors (inflammasomes), signaling pathways (NF-kB), and effector molecules (cytokines) and their direct anti-apoptotic effect on immune cells.

If a septic shock patient coming from the community does not show any signs of overwhelming shock nor of immune suppression the administration of a standard dose of 250 mg/Kg/day for 3–5 days should be considered after 12–24 h (Figure 2), when noradrenaline administration >0.1 mcg/Kg/min is persistently necessary to maintain target pressure and/or in case of a significant worsening of the CID score (60) indicating a non-positive evolution of the patient's status.

Hospitalized patients often have an impaired immune function due to multiple predisposing factors including multiple comorbidities, frequent exposition to antibiotics, high risk of MDR pathogens colonization/infection and, therefore, these patients should be a priori considered as immune-compromised and should be treated in the same way of immuno-suppressed patients coming from the community: within 6–12 h from shock occurrence, when noradrenaline dosages are higher than 0,1 mcg/Kg/min, at the standard dose of 250 mg/Kg/day for 3–5 days (Figure 2).

This simple protocol could help to roughly divide patients based on pre-existent conditions and shock characteristics waiting for further studies aimed to better clarify the efficacy of Igs administrations in this setting.

Alongside this suggested clinical protocol, we are running a multicenter RCT “Efficacy and Safety of Adjunctive IgM-enriched Immunoglobulin Therapy with a Personalized Dose Based on Serum IgM-titers vs. Standard Dose in Patients with Septic Shock,” which aims to compare whether a personalized dosage of IgGAM, to achieve and maintain serum titers above 100 mg/dl, could show a different impact on 28-day mortality than a standard dose (250 mg/kg for 3 days) *(NCT04182737)*.

## Conclusions

Septic shock requires further studies on the use of adjuvant therapies. To date, a process of selecting potentially profitable patients in terms of personalized medicine is preferable. To this purpose, we believe that a protocolized use of Igs therapy may be an option in the treatment of septic shock, however any type of protocol must be validated by a RCT before extensive clinical use.

## Author Contributions

SB, ER, MT, EB, and IC have written the initial draft. MM, SD, LB, RG, and MG reviewed the final version. ER, MT, EB, and IC also helped to draw the figures. All authors have written and reviewed the manuscript.

## Conflict of Interest

MG has consulted for Biotest-Germany. The remaining authors declare that the research was conducted in the absence of any commercial or financial relationships that could be construed as a potential conflict of interest.

## Footnotes

^1^Available online at: http://www.salute.gov.it/imgs/C_17_AvvisiSicurezza_8135_azione_itemAzione0_files_itemFiles2_fileAzione.pdf
